# Intraocular pressure improvement in patients receiving teprotumumab for the treatment of thyroid eye disease: a case series

**DOI:** 10.1186/s13256-022-03375-x

**Published:** 2022-05-10

**Authors:** Matthew Chu, Jonathan Sung, Michael Song, Alice Song, Julia Song

**Affiliations:** 1 Southern California Eye Physicians and Surgeons, 1111 S. Fair Oaks Ave., Pasadena, CA 91105 USA; 2 Center for Oculofacial and Orbital Surgery, 10861 Cherry St., #208, Los Alamitos, CA 90720 USA

**Keywords:** Teprotumumab, Thyroid eye disease, Intraocular pressure, Primary gaze, Lateral gaze, Upgaze

## Abstract

**Background:**

Teprotumumab is a novel treatment that reduces inflammation and symptoms caused by thyroid eye disease. There are limited data on teprotumumab’s effect on intraocular pressure.

**Case presentation:**

We report nine patients diagnosed with thyroid eye disease whose intraocular pressure decreased during teprotumumab treatment for 8 weeks: patient 1, a 67-year-old Hispanic woman; patient 2, an 86-year-old African-American man; patient 3, a 71-year-old Caucasian woman; patient 4, a 72-year-old Hispanic woman; patient 5, a 65-year-old Caucasian woman; patient 6, a 54-year-old Caucasian man; patient 7, a 54-year-old Asian man; patient 8, a 31-year-old Asian woman; patient 9, a 60-year-old Caucasian woman. The diagnosis of thyroid eye disease was based on increased redness, swelling, and excessive tearing; abnormal proptosis, lid retraction, and diplopia measurements were also taken during physical examination. Intraocular pressure in primary, lateral gaze, and upgaze was documented. There was significant (*p* = 0.0397) improvement of primary gaze eye pressure from pre-teprotumumab infusions (baseline) to completion of the treatment course.

**Conclusions:**

Teprotumumab significantly decreased the intraocular pressure for patients during the duration of the study. Teprotumumab is a novel medication that is approved for the primary treatment of thyroid eye disease in both acute and chronic thyroid eye disease. Previous treatments used to treat thyroid eye disease include glucocorticoids, radiotherapy, or orbital decompression surgery; however, these treatments all have significant limitations. Teprotumumab is an effective noninvasive alternative for decreasing symptoms of thyroid eye disease and, as shown, also lowers intraocular pressure. However, teprotumumab should not be used as a substitute for glaucoma medications; its ability to lower intraocular pressure may be in addition to lowering periorbital pressure and retro-orbital pressure.

## Introduction

Graves’ ophthalmopathy (GO), or thyroid eye disease (TED), occurs most often in patients with Graves’ disease, with a higher incidence in women and smokers. It is a potentially devastating condition that significantly impacts a patient’s quality of life [1]. TED typically begins with orbital and retro-orbital inflammation for 1–3 years, followed by a stable fibrotic period, which left untreated may lead to blindness or, more commonly, long-term proptosis or diplopia [2, 3]. The primary pathogenesis of TED is thought to be due to overstimulation of hyaluronan synthesis by GO fibroblasts [4, 5]. This leads to changes in, and enlargement of, extraocular muscles along with expanded orbital adipose tissue within the bony orbit increasing retro-orbital pressure [5]. The combination of these effects gives TED its typical presentation of proptosis and motility deficits. Patients commonly experience diplopia, blurred vision, redness, and pain. Patients can also experience eyelid retraction, strabismus, exposure keratopathy, and compressive neuropathy.

Teprotumumab inhibits the insulin growth factor 1 receptor (IGF-1R) complex [3, 4]. This complex interacts with thyrotropin receptors, which together are responsible for the upregulated immune response in TED [5]. Inhibition of the IGF-1R complex decreases the inflammatory cascade and upregulation of orbital fibroblasts and other immunologic factors responsible for adipogenesis in the orbit [3]. By targeting the IGF-1R protein, teprotumumab has been shown to significantly reduce proptosis, orbital fat volume, extraocular muscle volume, and diplopia, thereby improving patients’ quality of life [1]. There have been mostly mild and self-limiting adverse events documented, indicating a positive risk/benefit profile for teprotumumab in the treatment of TED.

Before teprotumumab, treatment options for TED included immediate cessation of smoking, glucocorticoid treatment, radiotherapy, and orbital decompression [6]. Clinicians have been treating the active stages of TED with immunosuppressant steroids in hopes of decreasing inflammation and scarring in the orbital region [6]. Steroids have been the standard treatment, with oral steroids having significantly less effect on reducing GO than intravenous administration [6]. Even with its effectiveness in reducing orbital inflammation, steroids do not halt TED’s progression and have no clinically appreciable effect on proptosis or diplopia [7]. After disease progression reaches the inactive phase, patients may continue to experience proptosis, diplopia, strabismus, dry eyes, elevated intraocular pressure (IOP), and, in the worst case, optic neuropathy. If indicated, surgical treatment through orbital decompression can improve overall symptoms along with the added effect of lowering primary gaze IOP; however, side effects are commonly postoperative numbness and diplopia [6, 7].

In addition to proptosis, motility changes, and inflammatory symptoms, patients with TED often present with elevated IOP, especially in upgaze [8, 9]. Elevated IOP in patients with TED occurs because of inferior rectus muscle restriction and decreased episcleral venous flow [9]. Limited data are available on the effect of teprotumumab on IOP. Here we present nine patients receiving teprotumumab who showed improvement in their TED symptoms and report primary gaze, upgaze, and lateral IOP changes throughout teprotumumab treatment.

## Methods

A sequential review of patient cases and of literature was performed. We recorded primary, upgaze, and medial gaze IOP to examine the improvement in IOP throughout treatment and conducted a paired two-tailed *t*-test, mean, standard deviation (SD), and percent change to determine whether the final IOP was significantly different from the baseline IOP. Applanation tonometry was used to measure IOP with Goldmann tonometry.

## Case presentation

The diagnosis of TED was based on physical examination of the patient. Patients reported redness, swelling, excessive tearing, and pain behind the eyes. Proptosis, lid retraction, and diplopia measurements were taken during the examinations. The standard dosage of teprotumumab (10 mg/kg on the first infusion and 20 mg/kg for the following infusions) was administered to each patient. A total of eight infusions were administered over 6 months every 3 weeks. Patient IOP is summarized in Table 1. The reported IOPs on patient presentation are the average between right eye (OD) and left eye (OS) IOP (pre-infusion and final infusion); these values were used to conduct the two-tailed paired *t*-test.

**Table 1:** Intraocular pressure (mmHg) throughout the 26-week trial of teprotumumab from baseline to study completion

Patient number	Baseline		1		2		3		4		5		6		7		8	
	OD	OS	OD	OS	OD	OS	OD	OS	OD	OS	OD	OS	OD	OS	OD	OS	OD	OS
1																		
Primary	12	14	10	10	9	13	10	10	12	9	13	13	9	10	12	12		
Lateral	9	12	12	11	12	14	13	12	14	10	13	13	13	13	12	12		
Upgaze	16	15	12	12	12	12	13	13	13	11	13	13	12	11	12	12		
2																		
Primary	15	17	11	12.5	15	15	15	15										
Lateral	15	14	11	11	17	17	17	16										
Upgaze	20	20	17	17	24	24	17	17										
3																		
Primary	20	20	19	19	14	15	13	10	14	15	17	18	15	15	15	15	17	17
Lateral	18.5	21	19	19	15	10	13	20	14	10	19	15	21	21	18	18	17	17
Upgaze	24	25	22.5	24	19	21	17	16	19	17	22	20	21	27	18	18	17	17
4																		
Primary	13	16	9	15	15	13	9	9	13	13	8	15	10	10	9	11		
Lateral	19	31	24	24	18	18	17	17	19	19	18	16	15	15	11	12		
Upgaze	38	56	28	28	26	14	17	17	19	19	15	18	18	18	11	16		
5																		
Primary	7	7	7	10	14	9	15	15	11	11	12	11	10	10	10	10	4	4
Lateral	7	7	6	6	12	12	15	15	17	17	12	11	10	10	10	10	4	4
Upgaze	5	5	14	11	12	10	15	15	11	11	12	11	10	10	10	10	4	4
6																		
Primary	12	13	10	13	9	10	10	10	10	10	9	12	12	14	10	10		
Lateral	12	12	12	15	13	14	15	15	17	13	21	19	14	14	10	13		
Upgaze	12	12	11	18	13	16	14	14	14	16	10	17	12	14	10	17		
7																		
Primary	22	22	23	22	17	13	21	16	17	17	18	15						
Lateral	27	29	26	26	17	17	19	18	23	23	22	22						
Upgaze	24	32	36	36	17	18	25	25	23	23	24	23						
8																		
Primary	11	13	16	14	13	13	12	4	17	15								
Lateral	13	13	19	17	16	22	9	10	22	20								
Upgaze	17	15	16	20	15	16	17	10	19	18								
9																		
Primary	17	17	11	15														
Lateral	12	12	17	17														
Upgaze	21	21	18	18														

We present upgaze and standard IOP measurements

Patient 1, a 67-year-old Hispanic woman, began with primary, lateral, and upgaze pressures of 13, 10.5, and 15.5 mmHg, respectively, and after completing all nine infusions had final IOPs of 12, 12, and 12 mmHg, respectively. There were improvements in primary and upgaze IOP but none in lateral gaze IOP.

Patient 2, an 86-year-old African-American man, had pre-infusion IOPs of 16, 14.5, and 20 mmHg. After completing three infusions of teprotumumab, the patient discontinued prior to the fourth infusion owing to uncontrolled blood sugar. His final IOPs were 15, 16.5, and 17 mmHg. There was an improvement in primary and upgaze IOPs.

Patient 3, a 71-year-old Caucasian woman, had pre-infusion IOPs of 20, 19.75, and 24.5 mmHg. After completing eight infusions, the IOPs were 17, 17, and 17 mmHg. The patient had been diagnosed with mild-to-moderate periorbital edema in the past. The patient showed improvement in all gaze pressures. The patient reported decreased pain and redness after completing infusions.

Patient 4, a 72-year-old Hispanic woman, had remarkable improvement after seven infusions were completed. Patient 4’s initial primary, lateral, and upgaze pressures were 14.5, 25, and 47 mmHg respectively, and after the seventh infusion were 10, 11.5, and 13.5 mmHg. She was concomitantly treated for primary open-angle glaucoma (POAG) and had been taking Xiidra twice a day (BID) both eyes (OU). Patient 4 continues to be treated for POAG, but reductions in swelling and pain behind the eyes after the final infusion were observed after treatment.

Patient 5, a 65-year-old Caucasian woman, had pre-infusion pressures of 7, 7, and 5 mmHg, and after eight infusions had final pressures of 4, 4, and 4 mmHg. There were decreases in IOPs for primary gaze, lateral gaze, and upgaze. She reported decreased pain and irritation after 3 months of treatment.

**Fig. 1:**
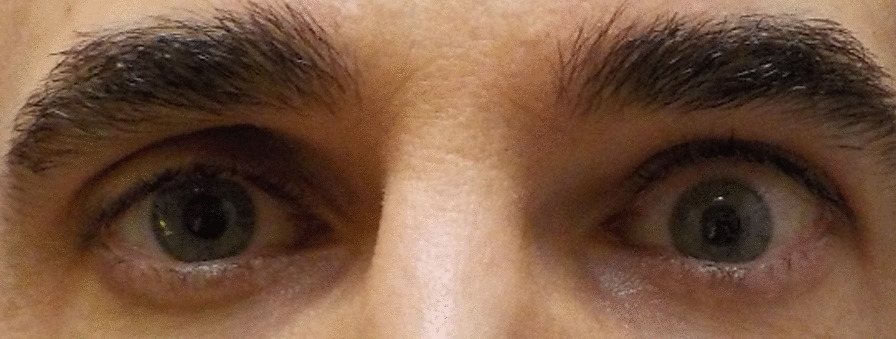
Patient 6 prior to teprotumumab treatment for TED

**Fig. 2:**
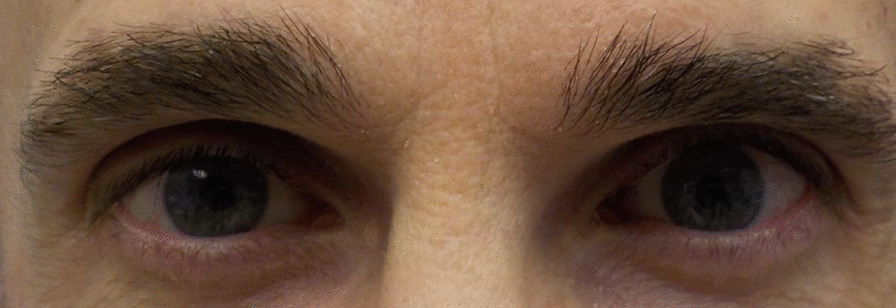
Patient 6 1 month after teprotumumab treatment for TED

**Fig. 3:**
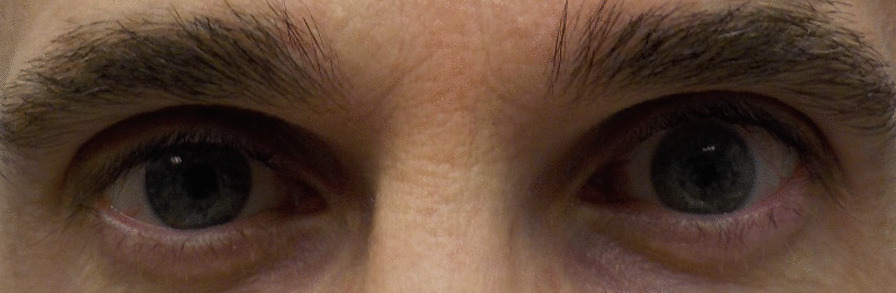
Patient 6 5 days after final teprotumumab treatment for TED

Patient 6, a 54-year-old Caucasian man, had pre-infusion pressures of 12.5, 12, and 12 mmHg and after completion had final pressures of 10, 11.5, and 13.5 mmHg. There was no change in primary gaze, a slight decrease in lateral gaze, and an increase in IOP in upgaze. Figure 1 displays patient 6 prior to teprotumumab treatment, Fig. 2 displays patient 6 1 month after starting teprotumumab treatment, and Fig. 3 displays patient 6 several days after the final teprotumumab treatment.

Patient 7, a 54-year-old Asian man, had pre-infusion pressures of 22, 28, and 28 mmHg. Patient 7 was also being treated for glaucoma and had been using Lumigan BID OU until the start of teprotumumab infusions. The patient had been diagnosed with acute orbital congestion in the past. The patient is currently undergoing his seventh infusion; however, the final pressures were 16.5, 22, and 23.5 mmHg. There had been a marked improvement in IOP in all gazes.

Patient 8, a 31-year-old Asian woman, had pre-infusion pressures of 12, 13, and 16 mmHg. Patient 8 is currently on her fifth infusion, and interestingly, IOPs have all increased with the most recent pressures of 16, 21, and 18.5 mmHg. The patient continues to be monitored as she continues treatment for TED.

**Fig. 4:**
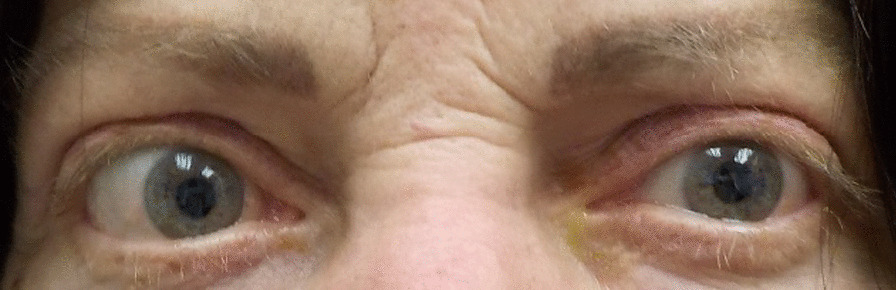
Patient 9 prior to teprotumumab treatment for TED

**Fig. 5:**
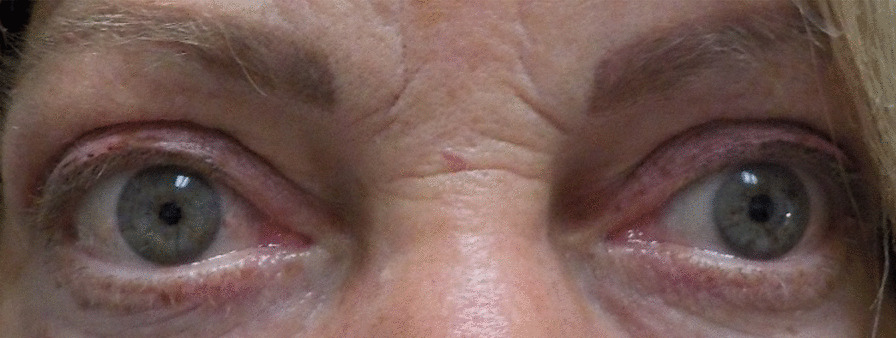
Patient 9 4 months after final teprotumumab treatment for TED

Patient 9, a 60-year-old Caucasian woman, had pre-infusion pressures of 17, 12, and 21 mmHg. We were unable to measure the IOPs during several post-infusion visits; however, she did return for her visit with final IOPs of 13, 17, and 18 mmHg. The patient demonstrated improvement in IOP in all gazes. Figure 4 displays patient 9 prior to teprotumumab treatment, and Fig. 5 displays patient 9 4 months after the final teprotumumab treatment; the patient reported experiencing less pain and irritation after completing treatment.

The baseline average IOPs in OD and OS in primary gaze were 14.89 mmHg (± 1.5). The baseline lateral IOP was 15.75 (± 2.3) mmHg, and the baseline upgaze IOP was 21.0 mmHg (± 4.0). The final IOPs in primary gaze, lateral gaze, and upgaze were 12.61 (± 1.4) mmHg, 14.72 (± 1.9) mmHg, and 15.22 (± 1.8) mmHg, respectively. The mean drop in IOP was −2.28 mmHg for primary gaze, −1.03 mmHg for lateral gaze, and −5.78 mmHg for upgaze. In primary gaze IOP, there was a 15.31% decrease in IOP from baseline and completion of the study (*p* < 0.05, Table 2). In lateral gaze, there was a 6.54% decrease from baseline to final IOP, yet this was not statistically significant (*p* > 0.05, Table 2). In upgaze, we observed a 27.52% decrease from baseline IOP; however, the decrease was not statistically significant (*p* > 0.05, Table 2).

**Table 2:** Primary and upgaze intraocular pressure change from baseline to completion of the study

Baseline primary IOP	14.89	± 1.501799332
Baseline lateral IOP	15.75	± 2.336307628
Baseline upgaze IOP	21.0	± 3.962497809
Final primary IOP	12.61	± 1.393879921
Final lateral IOP	14.72	± 1.859866914
Final upgaze IOP	15.22	± 1.808757844
Mean drop in IOP primary	−2.28	
Mean drop in IOP lateral	−1.03	
Mean drop in IOP upgaze	−5.78	
% decrease primary	15.31	
% decrease lateral	6.54	
% decrease upgaze	22.52	
*p* value primary	0.0397	
*p* value lateral	0.6394	
*p* value upgaze	0.1481	

IOP, intraocular pressure

## Discussion

A significant drop in IOP in primary gaze was observed from baseline to completion of teprotumumab treatment (Table 2). While the decreases in upgaze and lateral gaze IOPs were not significant across our limited sample, several of our patients individually improved throughout treatment. Furthermore, it was exciting to see a 22.52% decrease in upgaze IOP (Table 2). The mechanism by which teprotumumab lowers the IOP is not completely understood; it may be that the inhibition of the IGF-1R pathway and its subsequent decrease of extraocular muscle and adipose mass could have a significant role in improving episcleral venous flow. Additionally, teprotumumab may have decreased restriction in the inferior rectus and medial rectus muscles, which reduced in size during our study [1]. Lateral gaze IOPs in patients 1, 2, and 9 did not improve. This could be explained by enlarged medial and inferior rectus muscles. When the patient looks laterally, the enlarged medial rectus muscle may indent the globe and raise the IOP.

With positive results from the pivotal teprotumumab controlled trials combined with these findings of improved IOP with teprotumumab, it is exciting to see it becoming a standard treatment for TED. Additional studies need to be conducted to compare teprotumumab use against previous standard treatments for TED. This typically noninvasive option seems more viable for patients who are in the active, middle, and end stages of TED. Teprotumumab is a viable option unless there is a need for immediate surgical correction of optic neuropathy or corneal abrasion [10].

While teprotumumab is certainly an effective medication for the treatment of TED, the advent of targeted IGF-1R inhibition has its complications. A recent commentator discussed that appropriate forms of contraception should be implemented before initiation, during treatment, and 6 months following the last dose of teprotumumab due to its teratogenic properties [11]. Animal models have shown that long-term use of teprotumumab could be potentially harmful in pregnant patients and fetal development [11].

A number of patients in the current report had increased IOPs likely from glaucoma and TED, making our assessment of those patients more difficult. Patient 4, who had been concomitantly treated for glaucoma and TED, experienced a substantial decrease in IOP. However, the concomitant treatment of glaucoma medications and teprotumumab has not extensively been studied. Both oral and intravenous steroids used to treat early TED may not only initially treat inflammation but also adversely produce secondary steroid-induced glaucoma. In cases such as in patients 3 and 7, past medical diagnoses (mild-to-moderate edema and acute orbital congestion) may have influenced responses to teprotumumab; more research in this area is needed. While there may be some association between TED symptoms (inflammation) and glaucoma, none of the patients experienced visual field loss from elevated IOP. In addition, our sample size is limited to a smaller population of patients for up to 26 weeks. Future studies examining patients with glaucoma and TED should be conducted to assess the patient outcomes for managing both diseases.

## Conclusion

With the advent of teprotumumab and its positive effects on lowering IOP as a byproduct of lowering extraorbital muscle (EOM) and adipose mass, we are excited to see it being used as a primary medication for the treatment of TED. Our report documents nine patients who have significant improvement in primary gaze IOP and some who have an improvement in upgaze and lateral IOP. Teprotumumab’s ability to inhibit the IGF-1R complex and reduce orbital inflammation naturally reduces IOP. However, the statistically significant reductions in IOP we observed may have been due to differences in TED severity in each patient; reductions in enlarged extraocular muscles and orbital adipose tissue could have been greater in some patients than in others. For instance, patient 8 may not have responded to teprotumumab as well as patient 3 did. While there are some options for the treatment of TED, teprotumumab has shown to be an exciting development due to its efficacy, minimal invasiveness, and short duration of treatment. TED is complex, especially with added complications in patients with glaucoma. Thus, research focusing on the concomitant treatments of teprotumumab and other medications is necessary. We hope additional studies will be conducted to fully evaluate the use of teprotumumab in lowering IOP in patients who present with even more complex presentations.

## Authors’ information

The authors are located in the USA, involved in the medical field.

## Data Availability

Data and materials are available upon request.

## Abbreviations

OHT: Ocular hypertension
TED: Thyroid eye disease
GO: Graves’ ophthalmology
OS: Left eye
OD: Right eye
OU: Both eyes
BID: Twice a day
IOP: Intraocular pressure
IGF-1R: Insulin growth factor 1 receptor
OAG: Open-angle glaucoma
POAG: Primary open-angle glaucoma
SOAG: Secondary open-angle glaucoma
EOM: Extraorbital muscle

## References

[1] Douglas RS, Kahaly GJ, Patel A, *et al*. Teprotumumab for the treatment of active thyroid eye disease. N Engl J Med. 2020. 10.1056/NEJMoa1910434. 31971679

[2] Bahn RS. Graves’ ophthalmopathy. N Engl J Med. 2010;362(8):726–38. 10.1056/NEJMra0905750. PMC3902010 20181974

[3] Smith TJ, Hegedüs L. Graves’ disease. N Engl J Med. 2016;375(16):1552–65. 10.1056/NEJMra1510030. 27797318

[4] Pritchard J, Han R, Horst N, Cruikshank WW, Smith TJ. Immunoglobulin activation of T cell chemoattractant expression in fibroblasts from patients with Graves’ disease is mediated through the insulin-like growth factor I receptor pathway. J Immunol. 2003;170(12):6348–54. 10.4049/jimmunol.170.12.6348. 12794168

[5] Bahn R. Current insights into the pathogenesis of Graves’ ophthalmopathy. Horm Metab Res. 2015;47(10):773–8. 10.1055/s-0035-1555762. 26361262

[6] Verity DH, Rose GE. Acute thyroid eye disease (TED): principles of medical and surgical management. Eye. 2013;27(3):308–19. 10.1038/eye.2012.284. PMC3597885 23412559

[7] van Geest RJ, Sasim IV, Koppeschaar HPF, *et al*. Methylprednisolone pulse therapy for patients with moderately severe Graves’ orbitopathy: a prospective, randomized, placebo-controlled study. Eur J Endocrinol. 2008;158(2):229–37. 10.1530/EJE-07-0558. 18230831

[8] Danesh-Meyer HV, Savino PJ, Deramo V, Sergott RC, Smith AF. Intraocular pressure changes after treatment for Graves’ orbitopathy. Ophthalmology. 2001;108(1):145–50. 10.1016/S0161-6420(00)00477-2. 11150280

[9] Spierer A, Eisenstein Z. The role of increased intraocular pressure on upgaze in the assessment of Graves ophthalmopathy. Ophthalmology. 1991;98(10):1491–4. 10.1016/S0161-6420(91)32100-6. 1961632

[10] Nassr MA, Morris CL, Netland PA, Karcioglu ZA. Intraocular pressure change in orbital disease. Surv Ophthalmol. 2009;54(5):519–44. 10.1016/j.survophthal.2009.02.023. 19682621

[11] Ting M, Ezra DG. Teprotumumab: a disease modifying treatment for Graves’ orbitopathy. Thyroid Res. 2020. 10.1186/s13044-020-00086-7. PMC7334856 32636936

[12] Kim S, Patzek S. Teprotumumab for active thyroid eye disease. N Engl J Med. 2020;382(20):1958–9. 10.1056/NEJMc2002754. 32402170

